# Mirizzi syndrome masquerading as gallbladder carcinoma: a case report on diagnostic challenges and multidisciplinary management

**DOI:** 10.3389/fmed.2025.1586112

**Published:** 2025-06-02

**Authors:** Yu Yang, Yiwei Hou, Li Yi, Hang Zhou, Lihua Tang, Yunxi Fu, Mingzheng Hu, Rongchun Xing

**Affiliations:** ^1^The First College of Clinical Medical Science, China Three Gorges University, Yichang, China; ^2^Department of Hepatobiliary Surgery, Yichang Central People’s Hospital, Yichang, China; ^3^Department of Endocrinology, Yichang Central People’s Hospital, Yichang, China; ^4^Medical Technology College of Qiqihar Medical College, Qiqihar, China; ^5^Department of Pathology, Yichang Central People’s Hospital, Yichang, China

**Keywords:** Mirizzi syndrome, gallbladder cancer, case report, obstructive jaundice, cholangitis

## Abstract

**Background:**

Mirizzi syndrome is a rare condition that is frequently misdiagnosed as gallbladder cancer due to overlapping clinical and imaging features. This case underscores the diagnostic challenge of differentiating these two conditions and offers insights into managing such complex presentations.

**Case summary:**

A 45-year-old female presented with obstructive jaundice and elevated liver enzymes. Imaging studies, including CT, PET-CT, and MRCP, revealed gallbladder wall thickening, bile duct obstruction, and a high suspicion of malignancy. Tumor markers were within normal limits. Intraoperatively, extensive adhesions, gallbladder wall oedema, and bile duct compression consistent with Mirizzi syndrome were identified. A partial cholecystectomy and bile duct exploration with stent placement were performed. Histopathological examination confirmed chronic cholecystitis with inflammation, but no evidence of malignancy. The patient had an uneventful recovery, with complete resolution of her symptoms.

**Conclusion:**

Mirizzi syndrome can closely resemble gallbladder cancer, making careful surgical exploration and pathological evaluation essential for accurate diagnosis.

## 1 Introduction

Mirizzi syndrome is a rare biliary condition, accounting for 0.63 to 5.7% of all gallstone cases. It is characterized by the extrinsic compression of the bile duct caused by impacted gallstones in the neck of the gallbladder or the cystic duct (1, 2). Clinically, it manifests as obstructive jaundice, abdominal pain, and cholangitis, often resembling more severe pathologies, such as gallbladder cancer (3, 4). The overlap of clinical and radiological findings presents significant diagnostic challenges, particularly in patients with atypical presentations.

This case report highlights a unique presentation of a patient initially diagnosed with gallbladder cancer based on imaging studies, including computed tomography (CT) and positron emission tomography-computed tomography (PET-CT). However, intraoperative exploration and histopathological examination ultimately confirmed the diagnosis of Mirizzi syndrome. A literature review was conducted using the search terms “Mirizzi syndrome,” “gallbladder cancer,” “cholecystitis,” and “case report” in PubMed and Web of Science. This review revealed a limited number of cases in which Mirizzi syndrome mimicked gallbladder cancer (Table 1).

**Table 1:** Comparative analysis of Mirizzi syndrome cases mimicking gallbladder cancer: clinical, pathological, and therapeutic insights.

Cases	Prior clinical history	Clinical & imaging findings	Pathological evaluation	Mirizzi type / fistula	Tumor markers (↑/−)	Treatment approach	Follow-up / outcome
Mao et al. (25)	Biliary colic, weight loss	CT suggested gallbladder mass with a dilated biliary tree; MRCP suspected XGC and Mirizzi syndrome; malignancy could not be excluded.	Acute cholecystitis with empyema, no cancer.	I Mirizzi Syndrome (MS).	CA 19–9:210 kU/L (<35).	Subtotal fenestrating cholecystectomy was performed due to dense inflammation and empyema.	1 month/Full recovery, no malignancy.
Rizzo et al. (26)	Abdominal pain, jaundice	Ultrasound showed cholecystolithiasis; ERCP confirmed Mirizzi syndrome Type Va with cholecystobiliary and cholecystocolonic fistulas.	Chronic cholecystitis, no malignancy.	Mirizzi syndrome type V is complicated with both cholecystobiliary and cholecystocolic fistula.	-	Cholecystectomy, fistula takedown, biliary stenting, open biliary exploration with stone removal.	48 months/No recurrence.
Touati et al. (27)	Pruritus, jaundice	Imaging suggested Mirizzi Type II with a cholecystocholedochal fistula; MRI excluded malignancy.	Chronic inflammation with no malignancy.	Mirizzi syndrome type 2/cholecystocholedochal fistula.	-	Subtotal cholecystectomy, T-tube insertion for bile duct repair.	3 months/The Kehr drain was removed, and follow-up revealed no anomalies, with the patient in good health.
Zhou et al. (28)	Jaundice, abdominal pain	MRCP revealed hepatic duct stones and ascites; surgery found a fistula to the hepatic duct and left hepatic atrophy.	Porcelain gallbladder with chronic cholecystitis.	Mirizzi syndrome type 2/fistula of the common hepatic duct.	-	Cholecystectomy with bile duct repair for hepatic duct fistula.	Good recovery, no signs of malignancy
Chai & Xiao (29)	Chronic cholecystitis	Intraoperative findings revealed a biliary defect; preoperative imaging raised suspicion of malignancy.	Cholecystocholedochal fistula, no malignancy.	Mirizzi Syndrome Uncategorized	-	Open cholecystectomy with biliary defect repair using a gallbladder wall-free flap technique for high-risk patients.	48-60 months/Uneventful recovery

This table presents a comparative summary of five selected case reports of Mirizzi syndrome that closely resemble the current case. The comparison includes prior clinical history, diagnostic findings, pathological evaluations, Mirizzi type, tumor markers, treatment approaches, and patient outcomes. Each case underscores the diagnostic and therapeutic challenges in distinguishing Mirizzi syndrome from gallbladder cancer and highlights the critical role of comprehensive surgical exploration and histopathological evaluation in achieving an accurate diagnosis.

This report contributes to the understanding of a rare diagnostic dilemma and underscores the importance of a multidisciplinary approach in the evaluation and management of biliary diseases. By presenting this case, we aim to raise awareness among clinicians to consider Mirizzi syndrome as a differential diagnosis in patients with imaging findings suggestive of malignancy (1).

## 2 Case presentation

### 2.1 History and physical examination

A 45-year-old Asian female presented with progressive jaundice, dark-colored urine, and mild nausea persisting for 1 month. She reported no associated abdominal pain, fever, vomiting, or significant weight loss. There were no identified aggravating or relieving factors. Initial imaging at a local hospital indicated biliary obstruction, with a potential for malignancy. The patient was subsequently referred to our institution for further evaluation and management.

The patient’s history of present illness revealed no prior episodes of similar symptoms. There was no history of fever, chills, or right upper quadrant pain, which could suggest acute infection. She denied recent surgeries, hospitalizations, or travel. Her appetite and bowel habits remained unchanged. She had no family history of similar conditions, biliary disease, or cancer.

Her medical history was notable for chronic gastritis, for which she occasionally used over-the-counter proton pump inhibitors. She denied any history of diabetes, hypertension, or autoimmune diseases. Her surgical history was unremarkable, and she had no known drug allergies or adverse reactions. Socially, she was a non-smoker, did not consume alcohol, and worked as an office assistant. She reported no significant exposure to hepatotoxins or other occupational hazards. Family history was unremarkable for biliary or gastrointestinal cancers.

On physical examination, the patient appeared mildly jaundiced but was not in acute distress. Vital signs were stable, with no fever or tachycardia. Abdominal examination revealed mild tenderness in the right upper quadrant without rebound or guarding. Murphy’s sign was negative, and there was no palpable organomegaly, ascites, or lymphadenopathy. Systemic examination findings were otherwise normal.

### 2.2 Investigations

Laboratory assessment showed marked cholestasis—total bilirubin 6.5 mg/dL (0.1–1.2), direct bilirubin 5.2 mg/dL (0–0.4) and alkaline phosphatase 320 U/L (40–120)—with only mild transaminase elevations (AST 85 U/L, ALT 102 U/L). Inflammatory markers (WBC, CRP, procalcitonin), tumor markers (CA 19–9, CEA, AFP), renal function and electrolytes were all normal. See Table 2 for details.

**Table 2:** Longitudinal biochemical profile in Mirizzi syndrome management.

Project	2024/11/8	2024/11/15	2024/12/3	2024/12/11	2024/12/18	2025/1/22	Reference range	Unit of work
Alanine aminotransferase	253	39	54	47	14	14	7–40	U/L
Aspartate aminotransferase	305	39	49	45	13	19	13–35	U/L
Total bilirubin	172.8	127.8	44.5	53	23.4	11.2	0–23	umol/L
Direct bilirubin	113.7	79.4	27.1	33	10.9	3.6	0–6.8	umol/L
Alkaline phosphatase	563	352	154	-	204	101	42–98	U/L
Glutamyl transpeptidase	301	103	58	-	63	21	7–45	U/L
C reactive protein	4.31	-	3.61	-	-	2.34	0–6	mg/L
Alpha-fetoprotein	-	-	3.3	-	-	-	0–13.6	ng/ml
Carcinoembryonic protein	-	-	3.1	-	-	-	0–0.65	ng/ml
Carbohydrate antigen 199	-	-	2	-	-	-	0–39	U/ml
Carbohydrate antigen 724	-	-	2	-	-	-	0–6.9	IU/ml

Serial liver enzymes, bilirubin, and inflammatory markers (2024–2025) in a patient with Mirizzi syndrome. Values demonstrate initial hyperbilirubinemia and hepatobiliary dysfunction, with normalization post-intervention. Reference ranges and units are provided for comparison. Missing data (“-”) reflects unavailable results.

MRCP revealed multiple gallstones in the gallbladder and cystic duct, as well as a narrowed mid–common bile duct and upstream biliary dilatation. Contrast CT demonstrated significant gallbladder wall thickening and a hilar mass, while PET-CT showed increased uptake in the gallbladder and common bile duct, highly suggestive of gallbladder carcinoma.

**Figure 1:**
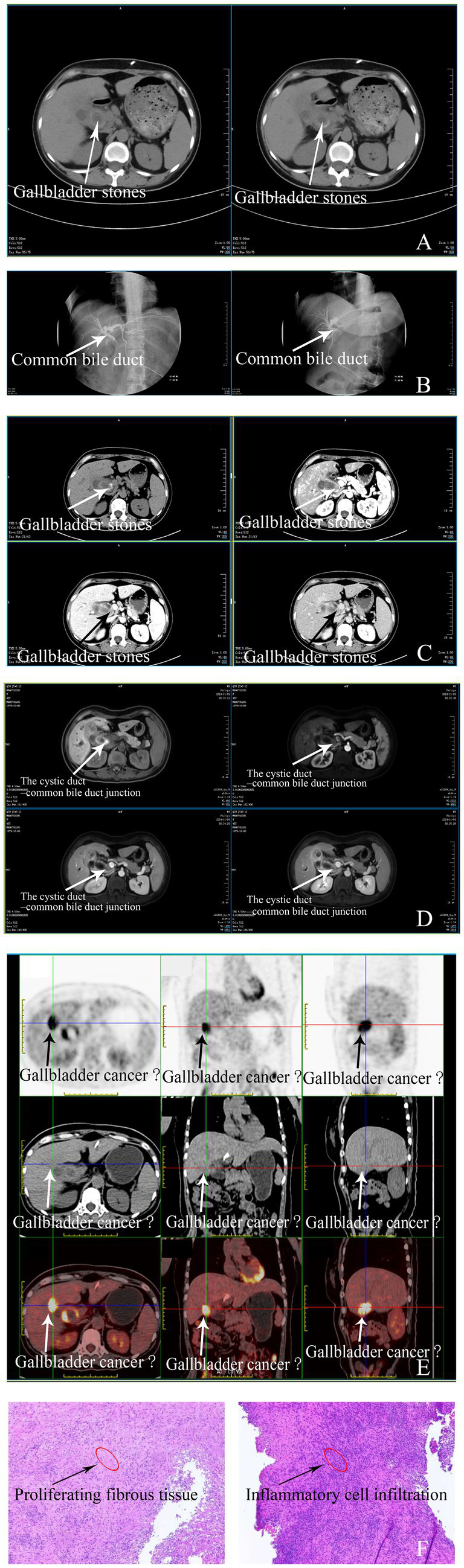
Imaging findings of gallbladder and related conditions. **(A)** Chest and Abdomen Plain CT: Lungs show increased markings and nodules. Post-PTCD, the liver is normal-shaped. The gallbladder is enlarged with high-density nodules, thickened walls, and dilated intrahepatic ducts. Retroperitoneal lymph nodes are present, and intrahepatic duct dilation is reduced compared to before. **(B)** PTCD Tube Contrast: Post-PTCD, ducts are dilated. The middle common bile duct has poor filling and slow contrast passage, with duodenal delay. **(C)** Liver Vascular Imaging: The liver looks normal. The gallbladder is enlarged with thickened walls (1.3 cm) and enhanced nodules. Vessels are normal, but the cystic artery is thickened, and there are retroperitoneal lymph nodes. **(D)** Abdomen MR: The liver has normal signals. The gallbladder has T2-nodular signals and a thickened wall (1.4 cm) with enhancement. The middle common bile duct is thickened, and stenosed, and intrahepatic ducts are dilated. Small retroperitoneal lymph nodes are seen. **(E)** PET Whole-body: The gallbladder and upper common bile duct have thickened walls with increased metabolism, likely malignancy. Gallbladder stones, lung fibrotic foci, and cervical cysts are also present. **(F)** Pathology Findings of Gallbladder Specimen: Microscopically, the suture-line marked area and the rest show proliferative fibrous tissue infiltrated by lymphocytes, plasma cells, etc. The biopsy lacks epithelial-lined gallbladder tissue, with no typical images of relevant diseases.

To alleviate jaundice, a percutaneous transhepatic cholangiography and drainage (PTCD) procedure was performed, which resulted in a reduction of bilirubin levels and an improvement in clinical symptoms. Percutaneous decompression was preferred over endoscopic retrograde cholangiopancreatography (ERCP) given the high-grade obstruction and altered biliary anatomy inherent to Mirizzi syndrome; percutaneous transhepatic biliary drainage affords more direct access for external drainage and has been shown to reduce the risk of post-procedural cholangitis and pancreatitis compared with ERCP in complex biliary obstructions (5). Given the high suspicion of malignancy, the patient subsequently underwent laparoscopic bile duct exploration, partial cholecystectomy, and bile duct stent placement. Although diagnostic laparoscopy could be considered initially to clarify uncertain findings, definitive surgical intervention was preferred directly in this case due to strong radiologic suspicion of malignancy and the potential risk associated with repeated surgical procedures (Figure 1).

### 2.3 Operative report

Intraoperatively, under general anesthesia, the patient was positioned supine, and the pneumoperitoneum was established through a sub-umbilical incision. Laparoscopic exploration revealed cholestatic discolouration of the liver, significant biliary dilation, omental adhesions surrounding the gallbladder, and severe gallbladder wall thickening with extensive oedema. Inflammation and adhesions obscured the gallbladder triangle structures. Blunt and sharp dissection were carefully performed to separate the gallbladder from adjacent tissues, including the omentum and duodenum.

Due to the extensive inflammation and unclear anatomical landmarks, partial cholecystectomy was carried out after decompressing the gallbladder, which released approximately 50 mL of bile. The gallbladder wall was thickened, and intraoperative frozen pathology ruled out malignancy. The cystic duct was dissected and found to be edematous and enlarged, with unclear boundaries at its confluence with the common bile duct, consistent with Mirizzi syndrome.

A cholangiocatheter was inserted into the common bile duct to further assess the biliary, revealing significant dilation of the common bile duct with multiple stones. These stones were carefully extracted, and the patency of the left and right hepatic ducts, as well as the sphincter of Oddi, was confirmed. Notably, the presence of a giant impacted stone at the gallbladder neck observed during dissection strongly suggested mechanical obstruction rather than neoplastic infiltration. Cholangioscopic exploration revealed free bile flow through the common bile duct without evidence of luminal narrowing or mass formation. These intraoperative findings, combined with frozen section analysis showing inflammatory changes but no malignant cells, allowed us to confidently exclude gallbladder carcinoma and proceed with partial cholecystectomy as definitive treatment for Mirizzi syndrome. A bile duct stent was placed to ensure proper drainage. Hemostasis of the liver bed was achieved using electrocautery, and two abdominal drains were placed in Winslow’s foramen and gallbladder bed. The abdominal cavity was thoroughly irrigated, and no active bleeding or bile leakage was observed. The trocar sites were closed after confirming proper hemostasis and removing all instruments.

The surgery was uneventful, with minimal intraoperative blood loss. The patient was returned to the ward in stable condition.

### 2.4 Postoperative stay and follow-up

**Figure 2:**
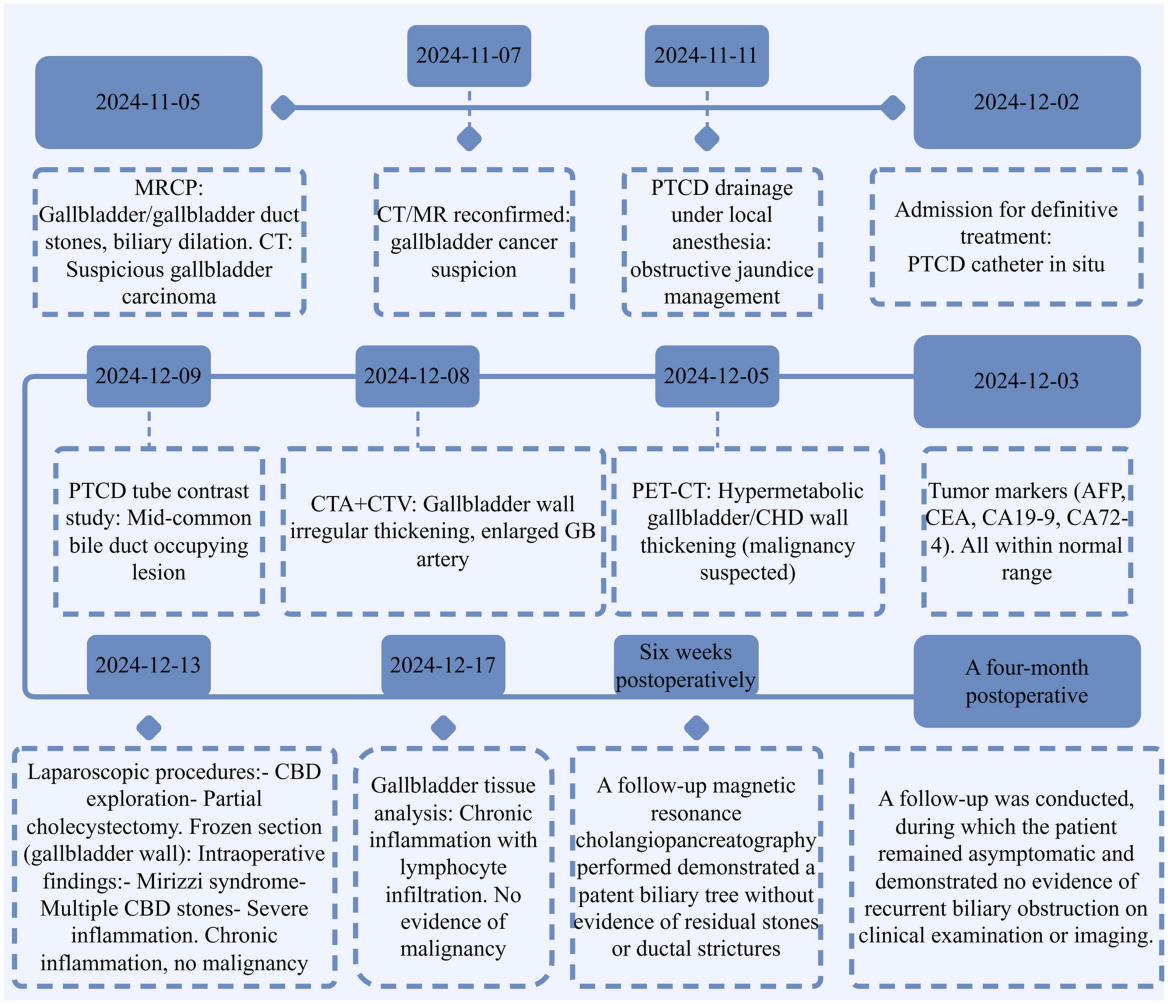
Timeline of key diagnostic and therapeutic events. This timeline highlights the diagnostic challenges in differentiating Mirizzi syndrome with secondary inflammation from gallbladder carcinoma, emphasizing the importance of multimodal evaluation and histopathological confirmation. Furthermore, it underscores the critical role of accurate diagnosis in determining patient prognosis, guiding appropriate therapeutic strategies, and influencing long-term follow-up and surveillance protocols.

Postoperative histopathology revealed chronic cholecystitis with acute cholangitis and significant inflammatory cell infiltration. Sections of the gallbladder wall demonstrated thickened fibrous tissue with infiltration of lymphocytes, plasma cells, and neutrophils, consistent with severe inflammation. No evidence of malignancy was identified, and there was no atypical epithelial proliferation or features suggestive of neoplasia (Figure 2). These findings confirmed the diagnosis of Mirizzi syndrome with inflammatory changes, excluding malignancy.

The patient recovered without complications and was discharged in stable condition. Specifically, a follow-up magnetic resonance cholangiopancreatography performed 6 weeks postoperatively demonstrated a patent biliary tree without evidence of residual stones or ductal strictures. A four-month postoperative follow-up was conducted, during which the patient remained asymptomatic and demonstrated no evidence of recurrent biliary obstruction on clinical examination or imaging. During follow-up consultations, the patient expressed gratitude for the multidisciplinary diagnostic approach and reported relief from the resolution of jaundice. She noted significant improvement in her quality of life postoperatively, highlighting restored strength and confidence in the treatment outcome. A detailed timeline is shown in Figure 2.

## 3 Discussion

This case highlights a rare instance of Mirizzi syndrome mimicking gallbladder cancer, a diagnostic challenge reported only a few times in the literature (6). Mirizzi syndrome typically presents with obstructive jaundice and bile duct dilation due to gallstones (2), but the imaging findings in this case were strikingly similar to those of gallbladder malignancy. Advanced imaging techniques such as PET-CT and MRCP revealed bile duct obstruction, gallbladder wall thickening, and increased metabolic activity, all of which strongly suggested malignancy (7). Relevant anatomical structures observed on imaging modalities, such as the gallbladder wall, cystic duct, and adjacent hepatic hilar structures, should be clearly highlighted to improve diagnostic accuracy. Additionally, histological images should distinctly illustrate inflammatory infiltrates, fibrosis, and epithelial integrity, which is crucial for distinguishing benign inflammatory conditions from malignancies. Furthermore, while serum tumor markers such as CA 19–9 and CEA are frequently employed to distinguish malignant from benign biliary disease, their specificity is limited: elevations can arise in benign obstructive jaundice and cholangitis due to cholangiocyte hyperplasia and inflammatory cytokine release, potentially yielding false-positive results (8). In contrast, assessment of acute-phase reactants—such as C-reactive protein and interleukin-6—may provide additional support for an inflammatory etiology when interpreted alongside imaging and histopathological findings (9). However, intraoperative findings and subsequent histopathology confirmed the diagnosis of Mirizzi syndrome, complicated by acute cholangitis and chronic cholecystitis.

Mirizzi syndrome is commonly diagnosed through a combination of clinical assessment, biochemical analysis, and advanced imaging modalities, including ultrasound, MRCP, and ERCP (10). Ultrasound typically serves as the initial screening tool, revealing gallstones and biliary dilation (11). MRCP and ERCP provide detailed visualization of biliary anatomy, pinpointing areas of extrinsic compression by impacted gallstones, thus confirming the diagnosis (12). Conversely, gallbladder cancer diagnosis relies significantly on imaging features suggestive of malignancy such as irregular gallbladder wall thickening, presence of intraluminal masses, and increased metabolic activity on PET-CT, supported ultimately by histopathological confirmation via biopsy or surgical specimen evaluation (13).

State-of-the-art management for Mirizzi syndrome involves surgical intervention tailored to the severity classified according to Csendes’ classification. Mild cases (type I) typically undergo laparoscopic or open cholecystectomy, whereas severe cases (types II–IV) require complex procedures including partial cholecystectomy, biliary duct reconstruction, and sometimes hepaticojejunostomy (14). Management of gallbladder cancer, however, is primarily surgical, entailing radical cholecystectomy with regional lymphadenectomy for localized disease, accompanied by chemotherapy or radiotherapy in advanced or metastatic cases (15).

The overlap in clinical presentation and imaging findings between Mirizzi syndrome and gallbladder cancer is well-documented but not fully understood. Literature reviews by Benchellal and others indicate that advanced cases of Mirizzi syndrome can cause inflammation-induced changes, such as wall thickening and fibrosis, which may mimic neoplastic processes (16). These findings emphasize the importance of correlating imaging results with intraoperative observations and histopathology to avoid unnecessary radical surgical procedures.

The uniqueness of this case lies in the extensive preoperative workup, which heavily favored a malignant diagnosis, including imaging findings and metabolic markers suggestive of cancer, despite normal tumor markers. Few cases have demonstrated such a marked discrepancy between imaging findings and histopathological results, highlighting the potential for inflammatory conditions like Mirizzi syndrome to produce false-positive imaging results for malignancy (17).

To enhance diagnostic accuracy, it is crucial to consider other conditions that may mimic or overlap with Mirizzi syndrome (18, 19). Differential diagnoses include gallbladder carcinoma, which may present with similar imaging findings such as wall thickening, biliary obstruction, and lymphadenopathy. Cholangiocarcinoma, particularly in the hilar region, can also cause bile duct stricture and proximal dilatation, necessitating histopathological confirmation (19). Primary sclerosing cholangitis may present with bile duct stricture and chronic inflammation but is often associated with multifocal biliary involvement and systemic conditions like inflammatory bowel disease (20). Choledocholithiasis with secondary biliary obstruction must also be considered, as large or impacted stones in the common bile duct can mimic the compression seen in Mirizzi syndrome (3, 19). Additionally, autoimmune cholangiopathy and IgG4-related sclerosing cholangitis can present with bile duct obstruction and wall thickening but can typically be differentiated through serological markers, imaging patterns, and response to steroid therapy (21, 22). Finally, infectious etiologies, such as biliary tuberculosis or parasitic infections like clonorchiasis, which cause bile duct thickening and obstruction, should be considered in endemic regions or in cases with corresponding clinical history (23).

Limitations of this case include the inability to fully evaluate the natural progression of the condition without surgical intervention, as well as the reliance on intraoperative findings and pathology as definitive diagnostic tools. These factors may limit the generalizability of the conclusions to cases managed without surgical exploration. Additionally, the rarity of this condition restricts the availability of comparative data in the literature.

A clear temporal and causal relationship was established between the patient’s symptoms, imaging findings, and intraoperative observations. The obstructive jaundice and inflammatory changes were directly attributed to bile duct compression caused by impacted gallstones in the gallbladder neck, consistent with Mirizzi syndrome (24). The absence of malignancy was confirmed through histopathological examination, further validating the accuracy of the diagnosis.

This case is significant due to the rarity of the presentation, the comprehensive diagnostic approach, and the successful surgical management. The findings were reviewed in the context of available literature and aligned with reported instances of Mirizzi syndrome mimicking malignancy, although with some unique features.

In conclusion, this case highlights the importance of considering Mirizzi syndrome in the differential diagnosis of biliary obstruction with features suggestive of malignancy. A multidisciplinary approach, incorporating detailed imaging, intraoperative exploration, and histopathological confirmation, is essential to avoid misdiagnosis and unnecessary radical interventions. Clinicians should remain cautious when interpreting imaging findings and conduct a thorough assessment of all potential differential diagnoses. Including conditions such as gallbladder carcinoma, cholangiocarcinoma, primary sclerosing cholangitis, autoimmune cholangiopathy, and infectious biliary disorders in the differential diagnosis is crucial for ensuring accurate and timely diagnosis. This case contributes to the growing awareness of the complexities in diagnosing and managing Mirizzi syndrome and underscores the need for ongoing research to improve diagnostic tools and strategies.

## 4 Conclusion

This case highlights the diagnostic challenge of differentiating Mirizzi syndrome from gallbladder cancer, emphasizing the need to combine imaging, intraoperative findings, and histopathological evaluation for an accurate diagnosis. Although imaging strongly suggested malignancy, Mirizzi syndrome was confirmed intraoperatively and by pathology, thus avoiding unnecessary radical surgery. Clinicians should maintain a high index of suspicion for benign conditions like Mirizzi syndrome in patients with obstructive jaundice and gallbladder wall thickening. Future research should focus on enhancing diagnostic accuracy through advanced imaging techniques and biomarkers to reduce misdiagnosis and optimize treatment strategies.

## Data Availability

The original contributions presented in the study are included in the article/Supplementary material, further inquiries can be directed to the corresponding authors.
